# An escalating dose study to assess the safety, tolerability and immunogenicity of a Herpes Simplex Virus DNA vaccine, COR-1

**DOI:** 10.1080/21645515.2016.1221872

**Published:** 2016-08-31

**Authors:** Julie L. Dutton, Wai-Ping Woo, Janin Chandra, Yan Xu, Bo Li, Neil Finlayson, Paul Griffin, Ian H. Frazer

**Affiliations:** a Admedus Vaccines Pty Ltd (formerly Coridon Pty Ltd), Translational Research Institute, Woolloongabba, QLD, Australia; b University of Queensland, Diamantina Institute, Translational Research Institute, Woolloongabba, QLD, Australia; c Q-Pharm Pty Ltd, Brisbane, Australia; Department of Medicine and Infectious Diseases, Mater Hospital and Mater Medical Research Institute, Brisbane, Australia; The University of Queensland, Brisbane, Australia

**Keywords:** codon-modification, DNA vaccine, Genital herpes, healthy volunteers, HSV-2, polynucleotide vaccine, ubiquitination

## Abstract

This paper describes a single site, open-label Phase I clinical trial evaluating the safety, tolerability and immunogenicity in healthy volunteers of a herpes simplex polynucleotide vaccine that has previously been shown to enhance immunogenicity and protect against lethal herpes simplex virus type 2 (HSV-2) challenge in mice. Five escalating doses of the vaccine, COR-1, were given by intradermal injection to HSV-1 and 2 seronegative healthy individuals. COR-1 was found to be safe and well-tolerated; the only vaccine-related adverse events were mild. While vaccine-induced antibody responses were not detectable, cell-mediated immune responses to HSV-specific peptide groups were identified in 19 of the 20 subjects who completed the study, and local inflammation at the immunisation site was observed. This study indicates COR-1 has potential to be used as a therapeutic vaccine for HSV-2 infection.

## Introduction

Genital herpes is a common sexually transmitted disease that results from infection of the genital mucosa with Herpes Simplex Virus type 2 (HSV-2) or, increasingly, by infection with HSV type 1 (HSV-1).^1-3^ While for some the infection is mild, others experience frequent and debilitating outbreaks. Rarely, HSV infection can lead to encephalitis in newborn babies, or ocular disease (such as herpes stromal keratitis) and HSV infection is believed to facilitate the transmission of Human Immunodeficiency Virus type 1.^4,5^ While antiviral medications are available to reduce the duration and severity of the outbreaks, these drugs are expensive, cannot eliminate outbreaks or shedding, and do not prevent recurrence of lesions and the spread of disease. Despite a number of clinical trials of potential vaccines for genital herpes, none have been shown to effectively prevent herpes infection.^6-8^ Chiron's recombinant gB2/ gD2 subunit vaccine formulated with MF59 adjuvant, despite generating neutralising antibodies, was ineffective in reducing HSV-2 acquisition.^9^ Another recombinant subunit vaccine, GSK's gD2/ alum/ 3-O-deacylated-monophosphoryl lipid A vaccine showed initial promise as, while it did not prevent HSV-2 acquisition in men or HSV-1 seropositive women, it did reduce HSV-2 disease by 70% and HSV-2 infection by 40% in HSV-1 and -2 double seronegative women.^10^ Unfortunately, this finding was not replicated in a larger follow-up study ^11^ and the vaccine was only shown to have an effect on HSV-1 disease and acquisition.

It is now understood that induction of high antibody titres alone is insufficient to prevent infection or the recurrence of lesions. Many studies indicate that cellular responses play an important role in preventing HSV-2 infection, reducing viral shedding, and producing a long-lasting memory response.^12,13^ This has been taken into consideration in the design of a therapeutic recombinant protein vaccine under development by Genocea Biosciences, GEN-003,^14^ and a polynucleotide vaccine by Vical.^15^ A Phase II dose optimisation trial of GEN-003 indicated that after 6 months the vaccine resulted in an up to 58 percent reduction in viral shedding and an up to 69 percent reduction in genital lesion rates, with 30–50% of patients lesion-free (unpublished; see press release http://ir.genocea.com/releasedetail.cfm?ReleaseID=935492). Vical's trial results have been less promising (http://www.vical.com/investors/news-releases/News-Release-Details/2015/Vical-Reports-Top-Line-Results-From-Phase-12-Trial-of-Therapeutic-Genital-Herpes-Vaccine/default.aspx). While there are live-attenuated vaccine candidates at various stages of development which could have prophylactic and/or therapeutic potential, they pose regulatory issues due to safety concerns.

In this study, we have carried out a single site, open label Phase I clinical trial of a HSV-2 polynucleotide vaccine, COR-1, that was designed to induce specific antibody and T cell responses upon intradermal (ID) delivery and has previously been shown to provide protection against HSV-2 challenge in a murine model.^16^ The vaccine was delivered ID as it has been established in animal studies that, in general, less DNA is required to induce immune responses when it is delivered ID than when it is delivered intramuscularly. This is probably due to the large concentration of relevant immune cells in the dermis relative to the muscle. Delivery of vaccine doses adequate to induce immune responses when delivered to muscle would not be achievable using a simple needle and syringe approach, other delivery methods and/or adjuvants would be required (e.g. electroporation).

Our vaccine uses a different approach to inducing a balanced immune response than those discussed above. COR-1 is a 1:1 mixture of 2 plasmids which carry codon-modified gene sequences that encode full-length glycoprotein D from HSV-2 (gD2) and ubiquitin-fused truncated gD2 and were optimized to generate an immune response to a polynucleotide vaccine delivered intradermally (ID) in mammals. The ubiquitin-encoding sequence was included to target the antigen to the proteasome for processing and to enhance cytotoxic T cell responses.

The primary objective of this initial trial was to examine the safety and tolerability of ID injection of 5 escalating doses of COR-1 in healthy individuals that were seronegative to HSV-1 and 2. Secondary and tertiary objectives of the study included determining whether the vaccine induces the production of anti-HSV gD2-specific antibodies and to provide information that may lead to the prediction of an optimised dose of COR-1 for induction of an efficacious immune response to protect against future HSV infection. Exploratory objectives were to determine whether resulting anti-HSV gD2 antibodies are neutralising and to determine whether COR-1 induces a cell-mediated immune response.

## Results

### Subjects

**Figure 1.**
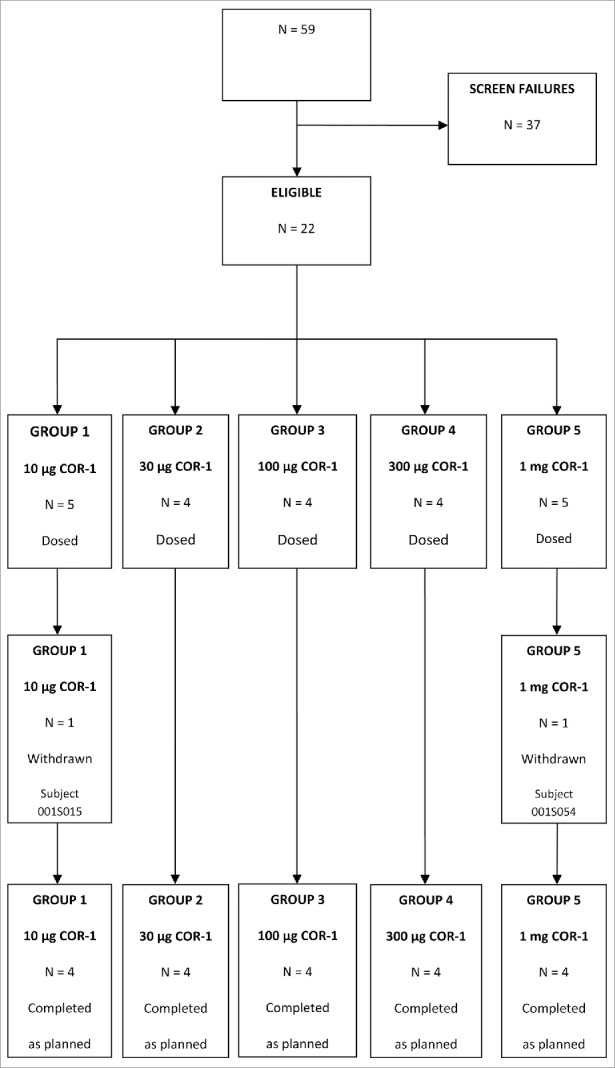
Disposition of subjects. N = number of subjects.

Study visits took place between August and December 2013. Fifty-nine potential subjects were screened for participation in the study, of these 22 were deemed eligible and 37 were not eligible for the study as they did not meet the inclusion criteria (i.e. HSV negative). Twenty subjects were enrolled in the study and 4 subjects were assigned to each of the treatment groups (Groups 1 to 5). Two subjects withdrew from the study after the first vaccine injection: one in the 10 µg COR-1 group (withdrew consent) and one in the 1 mg dose group (withdrew due to inability to comply with the protocol). Two replacement subjects were then enrolled and assigned to these groups. A total of 20 subjects completed the study as planned, 4 per treatment group. The disposition of subjects consented in the study is presented in Figure 1. Subject demographic and baseline characteristics are summarised in Table 1.

**Table 1.** Summary of demographic data.

COR-1						
	10 µg(N = 5)	30 µg(N = 4)	100 µg(N = 4)	300 µg(N = 4)	1 mg(N = 5)	Overall(N = 22)
Mean Age [years] (SD)	24.8 (5.4)	27.8 (4.3)	22.0 (2.2)	24.3 (1.5)	29.2 (3.3)	25.7 (4.3)
Gender						
Male	4 (80.0%)	1 (25.0%)	2 (50.0%)	1 (25.0%)	4 (80.0%)	12 (54.5%)
Female	1 (20.0%)	3 (75.0%)	2 (50.0%)	3 (75.0%)	1 (20.0%)	10 (45.5%)
Mean Height [cm] (SD)	181.2 (11.2)	170.9 (8.1)	174.8 (4.5)	167.2 (11.9)	175.9 (8.3)	174.4 (9.7)
Mean Weight [kg] (SD)	80.48 (16.51)	64.78 (10.34)	66.85 (9.04)	56.18 (9.14)	76.92 (14.49)	69.92 (14.56)
Mean BMI [kg/m^2^] (SD)	24.34 (2.53)	22.10 (2.31)	21.90 (2.08)	20.03 (1.01)	24.74 (3.40)	22.80 (2.85)
Race						
Asian	0	0	1 (25.0%)	1 (25.0%)	1 (20.0%)	3 (13.6%)
White	5 (100.0%)	4 (100.0%)	3 (75.0%)	3 (75.0%)	3 (60.0%)	18 (81.8%)
Other	0	0	0	0	1 (20.0%)	1 (4.5%)
Ethnicity						
Hispanic/Latino	0	0	0	0	1 (20.0%)	1 (4.5%)
Non-Hispanic/Latino	5 (100.0%)	4 (100.0%)	4 (100.0%)	4 (100.0%)	4 (80.0%)	21 (95.5%)
Alcohol Consumption						
Regular Drinker	4 (80.0%)	3 (75.0%)	3 (75.0%)	3 (75.0%)	2 (40.0%)	15 (68.2%)
Non-drinker	1 (20.0%)	1 (25.0%)	1 (25.0%)	1 (25.0%)	3 (60.0%)	7 (31.8%)

The treatment groups were relatively well-matched with regards to age, race and ethnicity (Table 2). The majority of subjects (80 %) were male in the 10 μg and 1 mg COR-1 treatment groups and the majority of subjects (75 %) in the 30 μg and 300 μg COR-1 groups were female. The 100 μg COR-1 group was composed of 50 % males and 50 % females. As a result, the mean height, weight and BMI was slightly higher for the 10 μg and 1 mg groups compared to the other treatment groups. The majority of subjects were white, non-hispanic and non-latino. Most subjects (75.0 % – 80.0 %) in the 10 μg, 30 μg, 100 μg and 300 μg groups were regular drinkers while 60.0 % of subjects in the 1 mg group were non-drinkers.

**Table 2.** Study schedule of events.

	SCREENING	VACCINATION									FOLLOW-UP
PHASE	Screening	Visit 1 1^st^ Vacc	Visit 2	Visit 3	Visit 4 2^nd^ Vacc	Visit 5	Visit 6	Visit 7 3^rd^ Vacc	Visit 8	Visit 9	Visit 10
VISITPROCEDURES	Day - 28 ±3 days	Day 0 ±3 days	Day 1 ±3 days	Day 2 ±3 days	Day 21 ±3 days	Day 22 ±3 days	Day 23 ±3 days	Day 42 ±3 days	Day 43 ±3 days	Day 44 ±3 days	Day 63/(ET)±3 days
Pre-selection and informed consent	√										
Saline injection to test for urticaria	√										
Demographics and medical history(incl. current medications)	√										
Drug history (incl. alcohol)	√										
Physical examination	√										√
Clinical laboratory tests^1^	√	√			√			√			√
HLA-typing^2^		√									
β-HCG ^7^ Serum-S/Urine -U	√ (S)	√ (U)			√ (U)			√ (U)			
Virology^3^	√										√ (HSV only)
Height and weight^4^	√	√			√			√			√
Inclusion/Exclusion criteria	√	√^5^									
Check-in		√			√			√			√
Vital signs^6^	√	√			√			√			√
Alcohol and drug screen	√	√			√			√			
Study vaccine administration and bleb checked		√			√			√			
Issue and collect diary cards		√	√	√	√	√	√	√	√	√	√
Review diary cards			√	√	√	√	√	√	√	√	√
Injection site examination^8^	√ (for suitability)	√	√	√	√	√	√	√	√	√	√
Adverse events and concomitant medications^9^		√	√	√	√	√	√	√	√	√	√
HSV Serology^10^		√			√			√			√
ELISPOT^11^		√			√			√			√
Check-out^12^		√			√			√			√

ET = Early Termination;

1) Samples for biochemistry and haematology panel collected at Screening, before each vaccination and on Day 63/ET (Visit 10).

2) Human Leukocyte Antigen (HLA) typing sample collected before vaccination on Day 0 (Visit 1) only.

3) Hepatitis B surface antigen (Hep B sAg), Hepatitis C (Hep C) and HIV, HSV 1 and HSV 2 serology. The Screening tests for HSV 1 and 2 could be conducted up to 60 days before the first vaccination.

4) Height and weight for Body Mass Index (BMI) at Screening. Weight check only performed at each clinical laboratory test visit to calculate creatinine clearance.

5) Exclusion criteria checked since previous visit before vaccination.

6) Blood pressure, radial heart rate, aural temperature and respiratory rate measured at Screening, before and 30 minutes after each vaccination and on Day 63/ET (Visit 10).

7) Urine β-Human chorionic gonadotropin (HCG) was negative before each vaccination.

8) Injection site examined, marked and photograph taken prior to each vaccination, 45 minutes, one day and 2 days after each vaccination and on Day 63/ET (Visit 10).

9) Adverse events and concomitant medications were recorded at check-in, at end of study evaluation and at any other time when spontaneously reported by a subject.

10) HSV Serology included HSV anti-gD2 antibodies by ELISA and anti-gD2 neutralising titres by PRNT_50_ collected 60 minutes prior to each vaccination and on Day 63/ET (Visit 10).

11) Blood sample for IFN-γ ELISPOT collected before 60 minutes prior to each vaccination and on Day 63/ET (Visit 10).

12) Checkout following completion of the visit assessments.

Medical history was reviewed at screening. Medical history that was ongoing at the start of the study included reactive airways disease (10 μg COR-1 group); eczema, asthma, hayfever and glucose-6-phosphate dehydrogenase deficiency (100 μg COR-1 group); acne and mild asthma (1 mg COR-1 group). None of these medical conditions excluded subjects from participation in the study.

A summary of the medications that were being taken prior to the study period and that were ongoing at the time of vaccination is provided in Table S2. Subject S009 (30 μg COR-1 group) took paracetamol for an upper respiratory tract infection following the Screening Visit but ceased this medication 6 days prior to the first vaccination.

### Safety and tolerability

Three ID injections of COR-1 were found to be safe and well-tolerated at the doses tested in this study, as assessed by vital signs, clinical laboratory evaluations, injection site examination, physical examination, and TEAEs.

A total of 35 TEAEs were reported during the study by 15 subjects (68.2%) (Table 3): the majority of these TEAEs were classified as mild and unlikely to be related to the vaccine. There was no relationship between the number of TEAEs and the vaccine dose for the 10ug to 300ug groups. However, the 1 mg group reported the most TEAEs, with 3 subjects reporting 10 cases of skin hyperpigmentation that were considered definitely related to the vaccine. Events reported by 2 subjects in the 10 µg COR-1 group were classified as moderate in severity but were not related to COR-1 (an influenza-like illness and an upper respiratory tract infection).

**Table 3.** Summary of treatment emergent adverse events.

	10 μg (N=5) Subjects (%) Events	30 μg (N=4) Subjects (%) Events	100 μg (N=4) Subjects (%) Events	300 μg (N=4) Subjects (%) Events	1 mg (N=5) Subjects (%) Events
At least one TEAE	5 (100.0%) 7	1 (25.0%) 2	3 (75.0%) 5	2 (50.0%) 2	4 (80.0%) 19
At least one Severe TEAE	0 (0.0%) 0	0 (0.0%) 0	0 (0.0%) 0	0 (0.0%) 0	1 (20.0%) 3
At least one Drug-Related AE[1]	3 (60.0%) 4	0 (0.0%) 0	1 (25.0%) 1	1 (25.0%) 1	4 (80.0%) 16
At least one TESAE	0 (0.0%) 0	0 (0.0%) 0	0 (0.0%) 0	0 (0.0%) 0	1 (20.0%) 1

[1] Drug-related is defined as unlikely, possibly, probably or definitely related to study drug.

Three events reported by one subject in the 1 mg COR-1 group were classified as severe. These included clavicle fracture, bone pain in the left clavicle and nausea, and were considered not related to the COR-1 vaccine, as they arose following a bicycle accident. This subject was given pain relief and nausea medication (paracetamol 1 g; oxycodone hydrochloride 5 mg; ondansetron 4 mg) following the accident. Further pain relief was prescribed (Ibuprofen 400 mg; oxycodone 20 mg) and the subject underwent surgery. This subject received the first and second COR-1 vaccinations as planned as these occurred prior to the accident. The subject received the third COR-1 vaccination 9 days after surgery, which was 3 days outside the time window specified in the protocol.

No subjects were withdrawn from the study due to TEAEs. No deaths or other significant TEAEs were reported.

**Figure 2.**
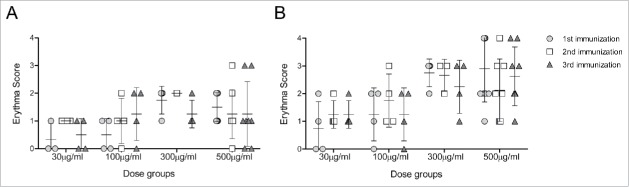
Injection site reactions. Scores corresponding to the injection site erythema size, measured 1 (A) and 2 (B) days after each vaccination, are plotted for subjects of cohorts that received 500 μg to 30 μg DNA vaccine. Note that the cohort which received 1 mg of DNA vaccine received 2 injections of 500 μg to both forearms. Scores of 1, 2, 3 and 4 were given for erythema measuring ∼0.5, ∼1.0, ∼1.5 and >1.5 cm, respectively.

A total of 143 local adverse drug reactions (ADRs) were reported during the study period by 21 subjects (95.5%). The number of local ADRs reported increased with increasing dose of COR-1. Erythema and induration were the most frequently reported injection site reactions, occurring in at least one subject in each treatment group at some point during the vaccination phase. In all treatment groups, any erythema reported had resolved by the next visit 3 weeks later. The incidence of induration tended to be greater in the 1 mg group (Fig. 2). All injection site reactions were classified as mild in intensity.

Four subjects (18.2%) reported a total of 14 systemic ADRs during the study period. The most frequently reported systemic ADRs were headache, fatigue, chills, malaise, pain and pyrexia. There were no trends between the frequency of systemic ADRs and treatment group. All the systemic ADRs reported were classified as mild in severity.

There were no changes over time in the haematology and biochemistry results or vital signs following vaccination with COR-1 at any dose level. In addition, COR-1 vaccination did not induce the production of anti-double stranded DNA antibodies.

### Immunogenicity

**Figure 3.**
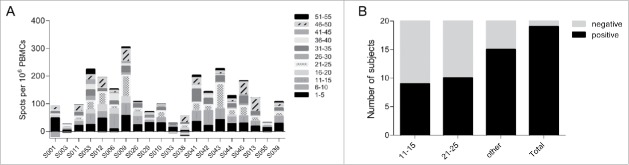
Pre-existing and vaccine-related cellular responses measured by IFN-γ ELISPOT. (A) Pre-existing responses to the gD2 peptide were determined using PBMCs taken at Visit 1 before the first vaccination. Responses were determined positive if X-(SD of X) – Y-(2xST of Y) > 10 (X: mean spot value; Y: mean spot value of no peptide control wells). (B) Number of subjects that were classified of having mounted a vaccine-related cellular immune response to gD2. Vaccine related positivity was accepted when the mean spot value was increased at least 2 fold compared to pre-existing responses in at least one peptide pool and at least one visit after vaccination.

No anti-HSV gD2 antibodies were detected in any subject by ELISA either before or after immunisation, indicating that no subjects had seroconverted to COR-1 during the study. In line with this, no neutralising antibody responses to HSV-2 were detected in any subjects. The incidence and size of post immunization erythema at the reaction site was greater in the 100 μg, 300 μg and 1 mg COR-1 treatment groups than in the lower dose treatment groups (Fig. 2). No erythema was detected in subjects receiving 10 μg COR-1. Furthermore, erythema sizes were greater 2 days after vaccination than 1 day, as would be expected with a delayed type sensitivity reaction. Interestingly, all but one subject had pre-existing cellular immune responses measured by IFN-γ ELISPOT to more than one peptide pool of gD2 despite being tested sero-negative (Fig. 3A). These responses were most commonly to peptide pools 11–15 and 21–25 (which contain known gD2 CD4 epitopes^17^ , and pools 1–5, 31–35 and 46–50. This implies that all subjects have potentially been in contact with HSV-2 and demonstrate memory cellular immune responses. We determined that a minimum of 2-fold increase in the mean spot value compared to pre-existing responses measured at Visit 1 in at least one peptide pool and at least one visit after vaccination would be regarded as a vaccine-related response. We concluded that 19 out of 20 patients had mounted a vaccine-related cellular immune response (Fig. 3B). Interestingly, a large proportion of subjects had responded to either peptide pools 11–15 or 21–25. We did however not observe a dose-dependent cellular response.

As measurable ELISPOT responses to HSV-2 peptides were seen in supposedly HSV naïve subjects, we further analyzed the gD2-specific ELISPOT data to establish the extent to which the observed increase or decrease in ELISPOT frequency post vaccination to each peptide pool was non-randomly biased toward an increase with vaccination (Table 4). As the range of changes were not Gaussian in distribution, with or without log transformation, the median change at each time point was assessed for each peptide pool, as well as the number of subjects for which an increase or decrease was seen. The results show that only peptide pool 21–25 induced significant change in ELISPOT count with immunisation across all peptide pools, and only at the first visit post immunisation (Table 4). 17 of 20 subjects responded positively to this peptide pool.

**Table 4.** Change in median ELISPOT count across all subjects, for each peptide pool, following immunization.

	post 1st immunisation			post 2nd immunisation			post 3rd immunisation		
Peptide pool	% change^a^	No. of Subjects negative	No. of Subjects positive^b^	% change^a^	No. of Subjects negative	No. of Subjects positive^b^	% change^a^	No. of Subjects negative	No. of Subjects positive^b^
1–5	−43	15	5	−9	11	9	−44	16	4
6–10	−27	13	7	31	12	8	−50	14	6
11–15	0	11	9	−3	8	12	−37	13	7
16–20	0	9	10	1	7	12	−22	11	7
21–25	107	3	17	9	11	9	25	8	12
26–30	53	5	13	31	10	10	42	8	12
31–35	−29	11	9	−16	11	9	−32	14	6
36–40	72	5	15	−15	12	7	−24	8	12
41–45	−31	10	9	−32	14	6	−40	11	9
46–50	−40	14	6	3	9	11	−57	16	4
51–55	−31	12	8	−25	13	7	−49	14	6

a % change in median spot count for this peptide pool from the pre-immunisation median.

b a positive subject showed a spot count more than 2 SD above the mean spot count for that subject compared to pre-immunisation.

## Discussion

A large number of DNA vaccines have undergone clinical trial and their general safety demonstrated.^18^ Consistent with those studies, this Phase I clinical trial clearly demonstrates that 3 intradermal immunizations with COR-1 is safe and well-tolerated in humans at doses up to 1 mg, with no vaccine-related moderate or severe TEAEs or SAEs observed. As erythema at the site of administration was mild, it is not a cause for concern. In addition, the vaccine did not generate anti-double-stranded DNA antibodies, which in theory could be a potential damaging side-effect of DNA vaccine administration, causing problems similar to those seen in systemic lupus erythematosus. The injection site hyperpigmentation observed in the highest dose group should fade over time and is also therefore unlikely to be a cause for concern.

Meeting a secondary goal of the study, the vaccine was shown to be immunogenic, giving rise to dose-dependent inflammation seen as erythema which was not due to the production of anti-dsDNA antibodies, and generating measurable gD2 antigen-specific cellular immune responses. The lack of anti-gD2 antibodies, in contrast to previous studies in mice,^16^ might reflect the relative low dose of vaccine given to subjects compared to the relatively high doses used in mice. The maximum dose of 1 mg roughly equates to a dose of 0.3 μg in mice. In a previous study, we showed that a high dose of 30 μg led to high and reproducible antibody titres in mice. When a low 0.3 μg dose was delivered twice, 2 weeks apart, to mice we found measurable levels of antibodies only in a minority of mice despite 70% of these mice surviving 50x LD_50_ HSV-2 viral challenge, indicating that clinical efficacy can be achieved with a low dose vaccination regime.^16^

It might also reflect the general difficulty of generating antibody responses to DNA vaccines in humans which has been noted in numerous clinical studies.^19-21^ Inflammation is rarely seen in response to DNA vaccine administration in humans, particularly if the vaccine is administered using a needle and syringe and no non-DNA boost, such as recombinant protein antigen or an adenoviral vector-based vaccine, is given.^20^ It is possible that the inflammation is in part due to the mode of delivery as a recent clinical trial comparing vaccine given IM and ID indicated that while ID resulted in more inflammation at the injection site than IM, the measured immunogenicity resulting from the 2 sites was similar.^22^ Of course, simply because the assays carried out in that study did not identify differences in humoral response does not mean there were none.

While it is disappointing that the vaccine did not generate detectable antibodies, it is remarkable that CTL responses were measured to at least one peptide pool in all but one of the subjects that received the full complement of vaccinations. As little as 10 μg of COR-1, delivered 3 times for a total dose of 30 μg, was sufficient to generate a measurable response. This result is of particular interest as, to our knowledge, needle and syringe delivery of a DNA vaccine has not consistently given rise to measurable cellular responses in humans at such a low dose. Few studies have examined the immune response to DNA vaccines delivered intradermally using a needle and syringe and without a recombinant protein or virus boost. Ledgerwood et al. ^23^ showed that 500 μg of plasmid DNA encoding an avian flu antigen, delivered 3 times, generated T cell responses in 20% of subjects. The only published studies which have delivered low doses (1–8 μg DNA) of plasmid DNA to the skin (and without protein/virus boosts) have used special delivery technologies, such as Particle Mediated Epidermal Delivery (PMED) ^24^ and are therefore not comparable with the present study.

The observed pre-existing responses to the HSV-2 gD peptides in all but one subject are interesting: while the subjects were all seronegative to HSV-1 and 2 by standard pathology testing and by further testing in a specialist HSV research laboratory, it seems likely that their immune systems had previously encountered the herpes simplex virus. It is possible that they were reacting to epitopes that just happen to be in the gD peptide sequences but are actually also present in another unrelated antigens. However, given that the responses were not restricted to one peptide pool, it seems unlikely that this is the case for all of the responses. This raises the possibility that the 19 subjects with pre-existing CTL responses are actually infected with HSV, most likely orofacial HSV-1, or that they have been exposed to, but have successfully resisted infection with or persistence of, the virus.

Immune correlates of protection for a HSV vaccine are not well-defined. Presumably, for COR-1 to be effective as a prophylactic it would need to be optimised in some way, such as by increasing the dose, or by modifying the dosing regimen or vaccine design, so that in addition to a cellular response, it induces the production of neutralising antibodies. Determination of an optimal dose of COR-1 to protect against future HSV infection is therefore unachievable from this study. However, the vaccine shows promise as a therapeutic vaccine as it is able to consistently generate cellular responses even at low doses. The generation of a T cell response is thought to be prerequisite to limiting recurring outbreaks, lesions and viral shedding, which could enhance life quality of infected individuals and also limit viral spread. This information, together with the safety data and the post hoc analysis of the injection site photographs, has been used to predict an optimised dose of COR-1 to potentially treat existing HSV infection. A blinded, placebo-controlled, dose escalation Phase I/IIa study of COR-1 in HSV-2 seropositive and symptomatic subjects that will primarily assess the safety and tolerability of the vaccine and its effect on viral load commenced in late 2015.

## Methods

### Subjects

Potential subjects were screened to assess their eligibility to enter the study. Subjects were eligible if HSV-1 and 2 seronegative; aged 18–45 years; generally healthy as determined by medical history with particular attention to drug history, chronic medication use, a review of body systems and physical examination findings; male or a non-pregnant, non-nursing female; if a female of childbearing potential the subject had to use 2 forms of highly effective birth control (failure rate < 1%/year) and to agree to continue to use this from enrolment through to study completion and for 4 months after the last vaccination; male subjects who were not surgically sterile used a condom from enrolment through to study completion and for 4 months after the last vaccination. In addition, the subjects had to have adequate venous access in their left or right arms to allow collection of a number of blood samples; have no birthmarks, tattoos, wounds or other skin conditions on both forearms which could reasonably obscure injection site reactions. Subjects also had to be able to communicate effectively with study personnel and be considered reliable, willing and cooperative in terms of compliance with the protocol requirements; not intend to start or change an existing physical conditioning regimen prior to or during the treatment period; and voluntarily give written informed consent to participate in this study.

Subjects who met any of the following criteria were excluded from participation in the study: suffered from saline-induced urticaria from the injection administered at the Screening Visit; had a current acute or chronic disease that would increase the expected risk of exposure to COR-1 or would be expected to interfere with the planned evaluations of response to COR-1, in the judgement of the Investigator (this included (a) an active medical condition that was under evaluation or treatment, or a recent illness, a chronic illness, an autoimmune disease or major surgery within the last year; (b) a history of clinically significant gastrointestinal, hepatic, renal, cardiovascular, dermatological, immunological, respiratory, endocrine, oncological, neurological, metabolic, psychiatric disease or haematological disorders; (c) a history of malignancy, other than non-melanoma skin cancer; (d) a history of cold sores or genital lesions or HSV infection; (e) a history of abnormal bleeding tendencies or thrombophlebitis unrelated to venepuncture or intravenous cannulation, or a history of Hepatitis B, Hepatitis C or HIV infection; (f) a history of allergy to any medication; (g) positive clinical laboratory serology for HSV, Hepatitis B surface antigen, Hepatitis C or HIV antibodies.); had a history of, or current evidence at the time of screening, of abuse of alcohol or any drug substance, licit or illicit, or current alcohol consumption was >4 standard drinks (or equivalent) per day; had received any vaccine or another investigational drug within 30 days prior to the Screening Visit or was due to receive subsequent vaccine boosters during the treatment period; was receiving chronic treatment with immune-suppressive therapy (asthma inhalers and topical corticosteroids were permitted); had a history of any psychiatric illness or psychological disorder which impaired the ability to provide written informed consent or participate in the study; had donated blood or plasma within 60 days prior to the Screening Visit; had participated in the past in another clinical trial of vaccination related to infection with HSV; was pregnant, nursing or had a positive serum β-HCG result at screening or at any time prior to subsequent vaccination with COR-1; had unusual dietary habits and excessive or unusual vitamin intake likely, in the opinion of the Investigator, to affect safety pathology parameters. All medications were documented and reviewed for acceptance by the Investigator or a medically-qualified nominee.

All subjects were HSV serology tested. Sufficient individuals were screened to recruit 20 subjects and 10 reserves.

All subjects provided informed consent prior to screening. The Queensland Institute of Medical Research (QIMR) Human Research Ethics Committee (HREC) approved the study, which was conducted in accordance with the Therapeutic Goods Administration (TGA) of Australia Note for Guidance on Good Clinical Practice and the Declaration of Helsinki.

### HSV serology testing

HSV-1 and HSV-2 serology testing was carried out by Sullivan Nicolaides Pathology (Brisbane, Australia) and the serum samples retested by the Centre for Infectious Diseases and Microbiology Laboratory Services, Westmead Hospital (Sydney, Australia) using the Enzygnost Anti-HSV/IgG (Siemens Healthcare Diagnostics Products GmbH, Marburg, Germany) and Captia Herpes Group IgG assays (Trinity Biotech, Bray, Ireland). Subjects were only included in the study if they were negative to all 3 tests.

### Study design

This was a single site, open-label study, evaluating 5 escalating doses of COR-1 vaccine conducted at Q-Pharm Pty Ltd in Brisbane, Australia. The study was divided into 3 phases: screening from Day -28 to Day 0 (Visit 1), vaccination from Day 0 (Visit 1) to Day 44 (Visit 9) and a follow-up phase at Day 63 (Visit 10).

This was not a randomized study. Following screening, subjects were sequentially allocated to one of 5 dose groups (4 per group). Each dose group received 3 ID injections of COR-1 at 3-weekly intervals. For each treatment group, one sentinel subject was vaccinated a minimum of 24 hours before the remaining 3 subjects in the group. Dose escalation to the next group proceeded no sooner than 24 hours after the last subject in the previous group had received their first vaccination. The study schedule of events is presented in Table 2.

### Study vaccine

The COR-1 vaccine was manufactured under Good Manufacturing Practice (GMP) conditions by VGXI Inc. (Texas, United States) under license from Admedus Vaccines Pty Ltd (formerly Coridon Pty Ltd), as previously described.^25^ The vaccine was supplied frozen at a concentration of 2.5 mg/mL to the investigation site. At the investigation site, the vials of vaccine were stored at -20 ± 5˚C in a secure area.

The COR-1 vaccine is a 1:1 mixture, by weight, of 2 DNA plasmids (COR-1A and COR-1B) formulated with 10 mM (hydroxymethyl) amino methane hydrochloric acid (Tris HCl) and 1 mM ethylenediaminetetraacetic acid (EDTA) pH 8. It was supplied as 1.5 mL aliquots in sterile glass vials sealed with Teflon-coated butyl stoppers and aluminium crimp caps.

Details of the design and synthesis of the plasmids have previously been reported.^16^ Briefly, the coding sequences were optimised according to a proprietary codon usage table which was designed to optimise the immune response to a polynucleotide vaccine delivered intradermally (ID) in mammals. The sequences of the vaccine inserts are identical to those in the sequences deposited in GenBank (accession numbers for gD2 and ubiquitin-fused truncated gD2 are JF304427 and JF715063, respectively). In the ubiquitin-fused gD2 insert, sequence encoding a single repeat of ubiquitin is directly upstream of and in-frame with gD2_25–331_ (which lacks the signal peptide- and transmembrane-encoding sequences).

All doses administered in the study were prepared by authorised staff in the investigation site pharmacy according to instructions provided by Admedus Vaccines Pty Ltd. The vaccine was diluted in sterile, isotonic saline suitable for injection to achieve the desired doses of 10 µg, 30 µg, 100 µg, and 300 µg. No dilution was required for the 1 mg dose. A volume of 0.2 mL of the required vaccine solution was then drawn-up in a 1 mL syringe for administration to subjects. The dispensed syringes were stored at room temperature (15 – 25°C) and were used within 24 hours of preparation.

Doses were selected based on data obtained in mice and on practical limitations of the delivery method: delivery of more than 1 mg by needle and syringe would not be feasible as that would require too many injections per immunisation or a higher concentration of DNA (which would be too viscous). It was necessary to include low doses to assess safety of the vaccine before proceeding to the 1 mg dose.

The lot number of COR-1 vaccine used in this study was COR-1.12.N013.

### Study treatment

COR-1 vaccine was administered by ID injection using 27 gauge needles to the forearm on days 1, 21 and 42. Subjects in groups 1, 2, 3 and 4 received a single 0.2 mL injection of 10, 30, 100 or 300 μg, respectively, of COR-1 on each immunisation day, administered to alternate forearms. The highest dose group, group 5, received two 0.2 mL injections, one in each forearm, of 500 μg COR-1 per immunisation day, resulting in a total dose of 1 mg.

The site of the injection was an area of skin 5 to 10 cm below the elbow joint. If there was any injection site reaction still apparent from the previous injection then a different site at least 5 cm away from the previous injection site was used.

Each dose was administered by authorised site staff experienced in ID injections. If injected correctly a “bleb” or “raised wheal” became visible in the skin. If no bleb appeared or the bleb leaked within 15 minutes of injection this was noted. Once injected, the subject kept their forearm extended for 10–15 minutes until the bleb resolved. During this time the diameter of the bleb was measured. The injection site was not covered with a dressing following each injection and subjects were instructed not to touch or scratch the site.

### Safety and tolerability assessments

Assessments were based on treatment-emergent changes in vital signs, at each visit and 30 minutes after each vaccination; treatment-emergent changes in clinical laboratory tests at specified intervals after vaccination; incidence and severity of local reactions (soreness, redness, induration, ecchymosis, edema, itching and paraesthesia) at the site of vaccination (including measurement of erythemas); incidence and severity of systemic reactions (fatigue, myalgia, malaise, fever, rigors, arthralgia, nausea, diarrhea, light headedness, dizziness, hypersensitivity and headache); and the incidence of treatment emergent adverse events (TEAEs). The nature and timing of these assessments are summarised in Table 2.

### Samples for immune function assays

Blood samples were collected before dosing treatment at baseline (day 0), on days 21 and 42 of treatment, and day 63 post-treatment period. The harvested sera were frozen and shipped to Charles River Laboratories for analysis. Peripheral blood mononuclear cells (PBMCs) were isolated by Ficoll density gradient centrifugation and frozen in liquid nitrogen until analysis.

### Double stranded DNA antibody testing

Final serum samples were tested for the presence of anti-double stranded DNA antibodies (QML Pathology, Brisbane).

### ELISA and neutralisation assay

Charles River Laboratories (Edinburgh) analyzed the serum samples for gD2-specific antibodies by ELISA. For this assay, plates were coated with an E.coli-expressed HSVgD2 recombinant protein (Reagent Proteins, Product Code GMN-333) which contains residues 266–394 of gD2 and a GST tag. The positive control for the ELISA was a pooled serum sample derived from consenting HSV-2 positive patients (HREC/12/QPAH/348). Human serum that was determined to be sero-negative for HSV-1 and -2 was used as the negative control and goat anti-human IgG (FC Fragment) Peroxidase (Sigma-Aldrich; Product Number A0170) was used as the secondary detection antibody. A plate-specific cut point was calculated based on the mean detector response of blank matrix negative control samples multiplied by a mean correction factor, and measured at an optical density between 0.613–0.775.

The neutralisation assay (plaque reduction neutralisation test, PRNT) was performed independently of the anti-gD2 ELISA. It was carried out in the laboratory of Paul Young at the University of Queensland. The PRNT was carried out in 96-well plates. The serum sample to be tested was serial diluted and mixed for 1 hour at 37°C with 2000 PFU/ml HSV-2 (HSV-2 BNE2013-1, strain HG52) to allow HSV-2 neutralising antibodies to react with the virus and limit its capacity to infect host cells. The serum/virus mixture was subsequently added to a confluent monolayer of Vero host cells (ATCC). The culture was incubated for 2 hours at 37°C before the inoculum was removed. Cells were overlayed with carboxymethyl cellulose (Sigma) to prevent the virus from spreading, and incubated for 24 hours at 37°C. Plaques were immune-stained with mouse anti-HSV-2 (Trinity Biotech) and secondary goat anti-mouse IRdye800 conjugated antibody (Rockland). The number of plaque forming units was determined using the Odyssey Imager (Li-Cor Biosciences).

### Interferon-**γ** enzyme-linked immunospot assay

T-cell responses to peptide pools (five 15 mer peptides per pool) spanning the whole length of the HSVgD2 protein were measured (in triplicate wells) using Interferon (IFN) γ enzyme-linked immunosorbent spot (ELISPOT) assay to analyze HSV-2 specific IFN-γ production by T cells.

Sterile 96-well plates (Millipore) were coated overnight at 4°C with monoclonal IFN-γ antibody (1-D1K, Mabtech). After coating, plates were washed once with complete RPMI and blocked for 2 hours with complete RPMI containing 10% fetal calf serum (FCS, Life Technologies). PBMCs were rested for 2 hours after thawing and then stimulated with medium alone and pools of HSV-gD2 overlapping peptides (Mimotope). The positive control was anti-human monoclonal antibody CD3-2 (Mabtech).

Cells were incubated with stimulants at 37°C and 5% CO_2_ for 16 to 20 hours. Plates were washed 6 times in PBS/0.05% Tween 20 (PBS-T). For detection, biotinylated detection monoclonal antibody (7-B6-1; Mabtech) in PBS-T/0.5% FCS was added, followed by horseradish peroxidise (HRP)-conjugated strepavidin (Mabtech) and DAB substrate (Sigma). Plates were dried and spots were quantitated using an AID-ELISpot Reader System (Autoimmun Diagnostika GmbH, Germany).

A response was considered positive when the mean of the spots counted in a particular peptide pool (X) minus the corresponding standard deviation (SD) was higher than 10, after the mean of the no peptide wells (Y) minus 2 times the SD was subtracted i.e., X-(SD of X) - Y-(2x SD of Y)> 10. This method combines the ‘empirical approach’ and recommendations from the Cancer Immunotherapy Consortium (formerly the Cancer Vaccine Consortium) http://www.cancerresearch.org/cic.

If a subject had pre-existing cellular immunity to HSV despite being sero-negative at screening, a 2-fold increase in the mean triplicate spot forming units (SFU)/well by or at Visit 10 compared to the Visit 1 result was considered treatment-related.

### Endpoints

The primary safety endpoints are listed in Table S1. The immunogenicity endpoints included an assessment of HSV serology and treatment emergent changes in anti-HSV-gD2 antibody titres by ELISA. The exploratory endpoints included treatment emergent changes in (a) anti-HSV-gD2 neutralizing titres by plaque reduction neutralization titres with a 50% endpoint (PRNT50) and (b) T-cell responses to HSV-gD2 antigen stimulation measured by ELISPOT assay.

### Independent review of data

INC Research (Melbourne, Australia) compiled the data and prepared the clinical trial report.

## Supplementary Material

Supplemental_Material.docx
