# Supplementary material for: An escalating dose study to assess the safety, tolerability and immunogenicity of a Herpes Simplex Virus DNA vaccine, COR-1
Source: Hum Vaccin Immunother. 2016 Aug 31;12(12):3079–88. doi: 10.1080/21645515.2016.1221872 (PMC5215501; doi:10.1080/21645515.2016.1221872)
Supplement: Supplemental_Material.docx [file khvi-12-12-1221872-s001.docx]

**Supplementary material**

Supplementary Table 1. Primary safety endpoints

| **Primary safety endpoint** | **Timing** |
| --- | --- |
| Incidence of treatment emergent adverse events (TEAEs) | through to Visit 10 |
| Treatment emergent changes in vital signs (blood pressure [systolic and diastolic], radial pulse rate, aural temperature and respiratory rate) | 30 minutes after each vaccination |
| Treatment emergent changes in vital signs | at each visit beginning at Visit 1 |
| Treatment emergent changes in clinical laboratory tests | at specified intervals after vaccination(s) |
| Incidence and severity of local reactions (soreness, redness, induration, ecchymosis, oedema, itching and paraesthesia) at the site of vaccination | from Visit 1 through Visit 10 |
| Incidence and severity of systemic reactions (fatigue, myalgia, malaise, fever, rigors, arthralgia, nausea, diarrhoea, light headedness, dizziness, hypersensitivity and headache) | from Visit 1 through Visit 10 |

Supplementary Table 2. Summary of Ongoing Prior Medications

| **Treatment Group** | **Subject Number** | **Medication** | **Indication** |
| --- | --- | --- | --- |
| 10 μg COR-1 | 001S011 | ETONOGESTREL | CONTRACEPTION |
|  |  | MULTIVITAMINS | GENERAL HEALTH WELLBEING |
| 30 μg COR-1 | 001S006 | INTRAUTERINE CONTRACEPTIVE DEVICE | CONTRACEPTION |
|  | 001S012 | ORAL CONTRACEPTIVE | CONTRACEPTION |
|  | 001S026 | ORAL CONTRACEPTIVE | CONTRACEPTION |
| 100 μg COR-1 | 001S010 | ORAL CONTRACEPTIVE | CONTRACEPTION |
|  | 001S029 | SERETIDE | ASTHMA |
|  | 001S033 | ORAL CONTRACEPTIVE | CONTRACEPTION |
| 300 μg COR-1 | 001S041 | INTRAUTERINE CONTRACEPTIVE DEVICE | CONTRACEPTION |
| 1 mg COR-1 | 001S039 | LORATADINE | HAYFEVER |
|  | 001S045 | MINOCYCLINE HYDROCHLORIDE | ACNE |
|  |  | IRON | GENERAL WELL BEING |
|  | 001S054 | HERBAL NOS W/MINERALS | WELLBEING |
|  | 001S055 | ORAL CONTRACEPTIVE | CONTRACEPTION |
